# Destructive Agencies

**Published:** 1857-07

**Authors:** 


### DESTRUCTIVE AGENCIES.

NEARLY one-fourth of all the deaths in Massachusetts during eighteen hundred and fifty-five were from consumption ;—the next greatest human destroyer was dysentery, commonly called *bloody flux.* Consumption is seated in the lungs,—dysentery is located about that portion of the bowels immediately under the stomach. Cough is the most universally observed symptom in consumption ; passing blood is the inseparable attendant of dysentery. The spark which kindles up consumptive disease is sudden changes in the temperature of the body from a heat above what is natural to one that is below. The most universal cause of dysentery is the breathing of a bad air between sunset and breakfast-time in warm weather.

The practical knowledge of these things, a possible and wise avoidance of them, would sweep from the list of human maladies the two deadliest of all diseases known to civilized life ; and yet, not one in a dozen can be induced to wisely guard against cooling-off too soon after exercise, or to avoid the breathing of an unwholesome atmosphere in warm weather, especially in August and September, with their hot days and cool nights. The result of this ignorance and inattention is, that the average of human life in the most intelligent State in the Union, and the thriftiest, does not exceed twenty-eight years, when it ought to exceed "*three score and ten!*"

